# Extensive Stray‐Dog Mauling as Major Contaminated Crush Trauma Causing Severe Rhabdomyolysis, Acute Kidney Injury, and Overt Disseminated Intravascular Coagulation: Recovery Without Renal Replacement Therapy

**DOI:** 10.1002/ccr3.73822

**Published:** 2026-10-30

**Authors:** Rafil A. Bshara, Lana Adel Atroshi, Zidan Mohammed Abdullah, Barhast I. Mohamed Salim, Gilles Prince

**Affiliations:** ^1^ Emergency Medicine and Trauma Azadi Teaching Hospital Duhok Iraq; ^2^ Center for Kidney Diseases, Duhok Directorate General of Health Duhok Iraq; ^3^ Department of Intensive Care Azadi Teaching Hospital Duhok Iraq; ^4^ Department of Radiology, School of Medicine University of Duhok Duhok Iraq; ^5^ Faculty of Medicine Saint Joseph University of Beirut Beirut Lebanon

**Keywords:** acute kidney injury, critical care, disseminated intravascular coagulation, dog bite, dog mauling, rhabdomyolysis

## Abstract

An adolescent male developed severe rhabdomyolysis, acute kidney injury, overt disseminated intravascular coagulation, and respiratory fluid overload after extensive stray‐dog mauling. The course reflected overlapping traumatic, inflammatory, and infectious mechanisms. He recovered without renal replacement therapy through source control, individualized antimicrobials, conservative renal support, and timely transition to de‐resuscitation.

## Key Clinical Message

Extensive dog mauling should be approached as major contaminated crush‐and‐tear trauma with simultaneous attention to deep infection. Early surveillance for rhabdomyolysis, acute kidney injury, and DIC, source control, and timely transition to individualized de‐resuscitation can permit recovery without dialysis when standard renal‐replacement indications remain absent.

## 1 Introduction

Dog‐bite wounds are common, but extensive dog mauling can represent major contaminated crush‐and‐tear soft‐tissue trauma rather than simple puncture injury. Such injuries combine deep tissue disruption with polymicrobial inoculation and require careful irrigation, debridement, infection assessment, and tetanus/rabies prophylaxis when indicated [1, 2]. Severe dog‐bite reports often emphasize infection‐associated complications, including sepsis, disseminated intravascular coagulation, acute kidney injury, dialysis, amputation, or death, whereas reports emphasizing the systemic consequences of traumatic muscle injury are less common [3]. We report an adolescent with extensive stray‐dog mauling complicated by severe rhabdomyolysis‐associated acute kidney injury, overt DIC, suspected necrotizing soft‐tissue infection, and fluid‐overload respiratory compromise, who recovered without renal replacement therapy. The case illustrated the current principles of management of rhabdomyolysis, DIC, fluid stewardship, antimicrobial therapy and rabies in a resource‐limited intensive care setting.

## 2 Case History/Examination

A previously healthy 17‐year‐old male presented with severe agitation and depressed level of consciousness after more than 10 stray‐dog bites. On arrival, he was in shock, with cool, mottled and pale peripheries, delayed capillary refill, and thready weak peripheral pulses. He was tachypneic, with a blood pressure of 95/55 mmHg, heart rate of 125 beats/min, and oxygen saturation of 95% on room air. Initial arterial blood gas showed lactate 5.2 mmol/L and base excess −7 mmol/L, indicating significant tissue hypoperfusion. Baseline complete blood count showed a white blood cell count of 16.3 × 10^9^/L, hemoglobin of 15.0 g/dL, and platelet count of 136 × 10^9^/L. He had extensive contaminated wounds involving the scalp, trunk, and extremities, including two large irregular full‐thickness scalp lacerations with partial flap avulsion/degloving and numerous puncture wounds, abrasions, contusions, and irregular lacerations over the limbs and trunk.

Two large‐bore peripheral intravenous cannulas were inserted. Because of active heavy bleeding from the extensive scalp avulsion wounds together with the signs of shock already described, the major hemorrhage protocol was activated and warmed crystalloid, packed red blood cells, and tranexamic acid 1 g were administered. The normal initial hemoglobin was not used to exclude acute blood loss; it fell to 13.1 g/dL within hours and to 9.8 g/dL by Day 1, then reached a nadir of 8.0 g/dL over the following 48–72 h in the context of ongoing wound losses, crystalloid hemodilution, and developing DIC before stabilizing after packed red cell transfusion. Bedside extended focused assessment with sonography for trauma (eFAST) showed no intra‐abdominal free fluid, bilateral lung sliding, and no hemothorax. Rapid sequence intubation was performed for airway protection because of altered consciousness, progressive agitation, shock, and respiratory distress during ongoing trauma resuscitation. Computed tomography of the head showed no intracranial hemorrhage or skull fracture.

The wounds were irrigated, debrided, selectively approximated where feasible, and managed with serial wound care. Human rabies immunoglobulin 20 IU/kg was infiltrated into and around accessible wounds as anatomically feasible, with the remaining calculated dose administered intramuscularly distant from the vaccine site; rabies vaccination was started on Day 0 in accordance with WHO recommendations [2]. Tetanus prophylaxis was administered according to local protocol. Given shock, extensive contamination, and concern for deep soft‐tissue infection, empirical intravenous meropenem and vancomycin were initiated at admission according to local intensive‐care protocol [1]. Because hypotension and signs of impaired perfusion persisted despite initial crystalloid and blood‐product resuscitation, noradrenaline was started to maintain a mean arterial pressure of at least 65 mmHg and support end‐organ perfusion; it was discontinued the following day after hemodynamic stabilization. On hospital Day 1, the patient was alert and following commands, with adequate oxygenation on minimal ventilator settings and intact cough and gag reflexes. He passed a spontaneous breathing trial and was extubated without immediate complications. Baseline renal function was normal, with only mild transaminitis.

## 3 Investigations and Treatment

On Day 1, urine became dark brown, serum creatinine rose to 1.8 mg/dL, creatine kinase was 38,949 U/L, procalcitonin was 74.7 ng/mL, and AST/ALT rose to 571/190 U/L. Severe rhabdomyolysis‐associated acute kidney injury was diagnosed. The patient developed a marked early systemic inflammatory response. The white blood cell count rose from 16.3 × 10^9^/L on admission to 22.9 × 10^9^/L on Day 1 with 86% neutrophils (absolute neutrophil count approximately 19.7 × 10^9^/L), then fell to 11.6 × 10^9^/L on Day 2 and normalized to 7.7 × 10^9^/L by Day 3. Procalcitonin exceeded 100 ng/mL and C‐reactive protein reached 140 mg/L on Day 2, followed by a progressive decline in the setting of antimicrobial therapy and surgical source control. The systemic inflammatory response and AKI were therefore managed as multifactorial, with empirical antimicrobial therapy and source control targeting a possible bacterial contribution. In accordance with the AAST rhabdomyolysis consensus document, lactated Ringer's solution was infused at 150–200 mL/h while urine output remained around 150 mL/h, with serial monitoring of renal function, electrolytes, urine output, and volume status [4, 5, 6].

By Day 3, creatine kinase peaked at 53,960 U/L and creatinine at 3.48 mg/dL. Coagulopathy progressed to overt DIC (International Society on Thrombosis and Hemostasis [ISTH] score = 6: platelets 63 × 10^9^/L; D‐dimer 4528; PT 23 s; fibrinogen > 100 mg/dL) [7, 8]. The DIC pattern evolved dynamically, with the ISTH score peaking at 7 on Day 5, corresponding with a platelet nadir of 18 × 10^9^/L. No life‐threatening bleeding occurred. One adult therapeutic platelet dose was transfused because the severe thrombocytopenia, extensive traumatic wounds, and operative‐site bleeding risk created a clinical indication for platelet support; the platelet count rose to 70 × 10^9^/L. Despite D‐dimer peaking later at 29,206 ng/mL on Day 8, therapeutic anticoagulation was withheld because there was no clinical or radiological thrombosis and bleeding risk remained high, consistent with ISTH and British Committee for Standards in Hematology DIC guidance [7, 8]. The temporal relationships among rhabdomyolysis‐associated AKI, systemic inflammation, and DIC are summarized in Figure 1.

**FIGURE 1:**
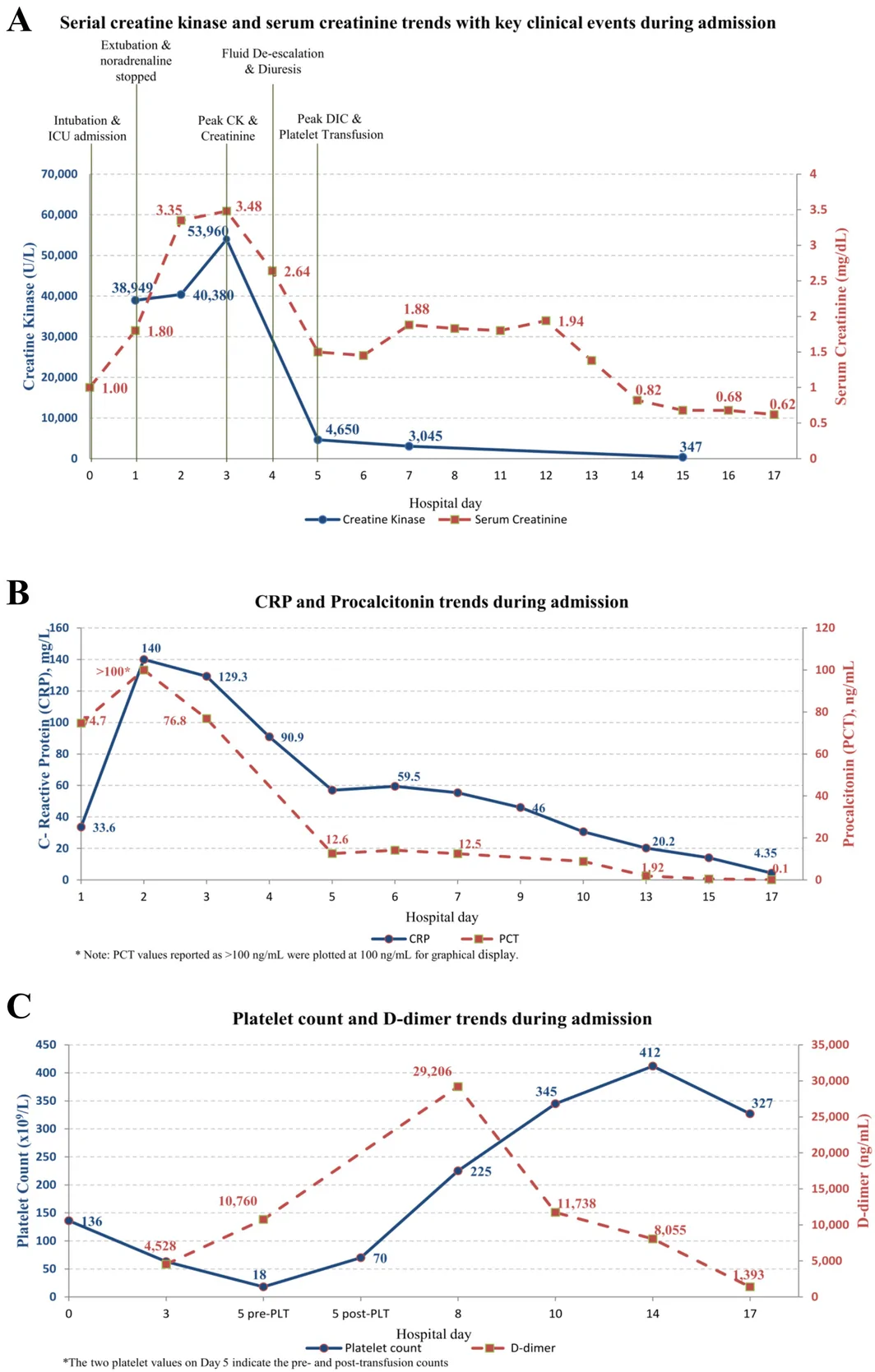
Clinical, biochemical, and hematological trends during the patient's intensive care admission. (A) Serial trends of creatine kinase (CK) and serum creatinine, illustrating severe rhabdomyolysis and acute kidney injury peaking concurrently on hospital Day 3. Vertical lines denote key clinical milestones, including initial resuscitation, recognition of fluid overload on Day 3, initiation of diuresis on Day 4, and the subsequent hematological crisis. (B) Inflammatory markers C‐reactive protein (CRP) and procalcitonin (PCT). Both markers peaked early on Day 2 and subsequently declined during antimicrobial therapy and source control, consistent with a mixed inflammatory response after extensive contaminated crush trauma. PCT values reported as > 100 ng/mL are plotted at 100 ng/mL for graphical display. (C) Coagulation profile demonstrating the dynamic evolution of overt disseminated intravascular coagulation (DIC). Platelet counts reached a nadir of 18 × 10^9^/L on Day 5, prompting a targeted platelet transfusion (shown as pre‐ and post‐transfusion values), while D‐dimer peaked later on Day 8 before gradually resolving. CRP, C‐reactive protein; DIC, disseminated intravascular coagulation; ICU, intensive care unit; PCT, procalcitonin; PLT, platelets.

On hospital Day 3, at the peak of the biochemical indices, the patient developed dyspnea with a respiratory rate of 30 breaths/min and oxygen saturation of 88% on room air. Arterial blood gas showed: pH 7.42, PaO_2_ 65 mmHg, PaCO_2_ 28 mmHg, $\mathrm{HC}O_{3}^{-}$ 23 mEq/L, with bilateral basal crackles on auscultation. Supplemental oxygen at 4 L/min by nasal cannula maintained oxygen saturation at 94%–95%. Electrocardiography and echocardiography were unremarkable. Lung ultrasound showed B‐lines, and bedside imaging demonstrated pleural effusion, ascites, a noncollapsing inferior vena cava (Supplementary Figures 1 and 2), and dependent scrotal oedema. NT‐proBNP was elevated to 5941 pg/mL, interpreted in the context of AKI, systemic inflammation, and positive fluid balance rather than primary structural heart failure [9]. Fluid overload was therefore recognized on Day 3, and noninvasive ventilatory support (CPAP/PSV) was initiated for work of breathing and hypoxaemia. Intravenous fluids were reduced to 50 mL/h and loop‐diuretic therapy with adjunctive albumin was commenced on Day 4 in alignment with contemporary de‐resuscitation recommendations [10]. Urine output increased to 200–250 mL/h over the following 24–48 h, achieving a net negative fluid balance of approximately 2.5–3.5 L/day. Oxygen requirement progressively decreased; supplemental oxygen was no longer required by Day 6, and the respiratory compromise resolved completely by Day 8 with a marked reduction in pleural effusions and ascites on repeat imaging.

Concurrently, worsening lower‐limb pain and foul wound odor raised concern for necrotizing soft‐tissue infection. Urgent MRI demonstrated diffuse bilateral subcutaneous oedema and small localized collections at bite sites (Figure 2). Urgent surgical exploration revealed no fascial necrosis, no loss of fascial resistance to blunt dissection, and no “dishwater” fluid; instead, contaminated inflammatory soft‐tissue wounds with localized collections were found. Wounds were opened or extended as needed, explored with curettage, thoroughly irrigated, and debrided. Vancomycin was discontinued within 24 h of admission as AKI evolved to limit additional nephrotoxic exposure; therapeutic drug monitoring was unavailable in the unit. Meropenem was continued with serial renal‐dose adjustment to maintain broad polymicrobial coverage. On hospital Day 8, after operative exclusion of necrotizing soft‐tissue infection, adequate source control, hemodynamic stabilization, and sustained improvement in inflammatory markers, antimicrobial therapy was de‐escalated to intravenous ceftriaxone plus metronidazole, which was continued to complete a total 14‐day antimicrobial course. The clinical wound evolution is shown in Figure 3.

**FIGURE 2:**
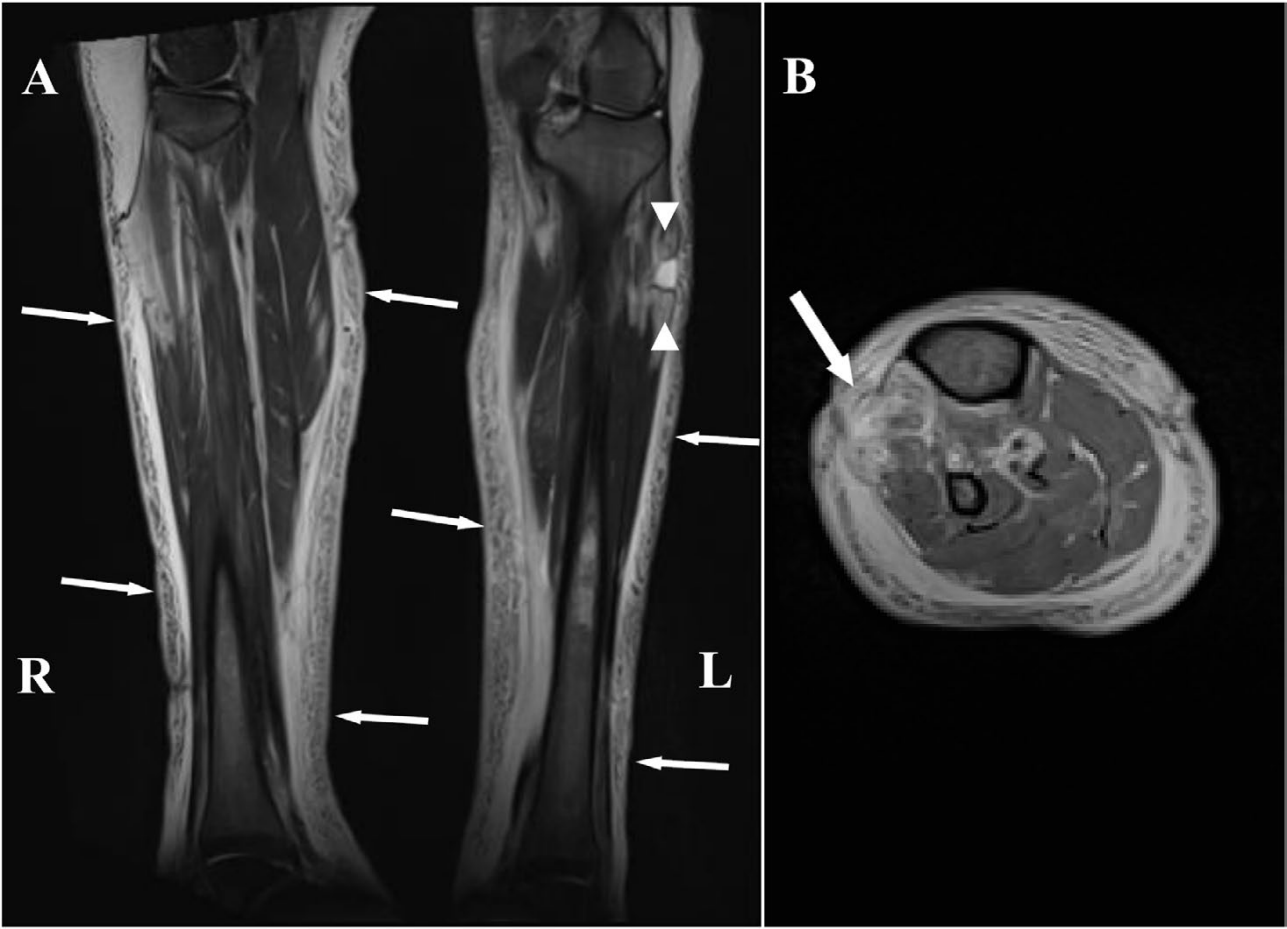
Magnetic resonance imaging of the lower limbs during evaluation for suspected necrotizing soft‐tissue infection. (A) T2‐weighted STIR coronal image of both legs showing diffuse bilateral subcutaneous soft‐tissue oedema (arrows). A bite site is also seen in the upper left leg as a skin surface break with a small localized subcutaneous collection (arrowheads). R and L denote the patient's right and left legs, respectively. (B) PD fat‐saturated axial image through the upper part of the right leg showing a bite‐site skin break with an underlying small localized soft‐tissue collection (arrows). Surgical exploration subsequently demonstrated contaminated inflammatory soft‐tissue wounds and localized collections, without fascial necrosis.

**FIGURE 3:**
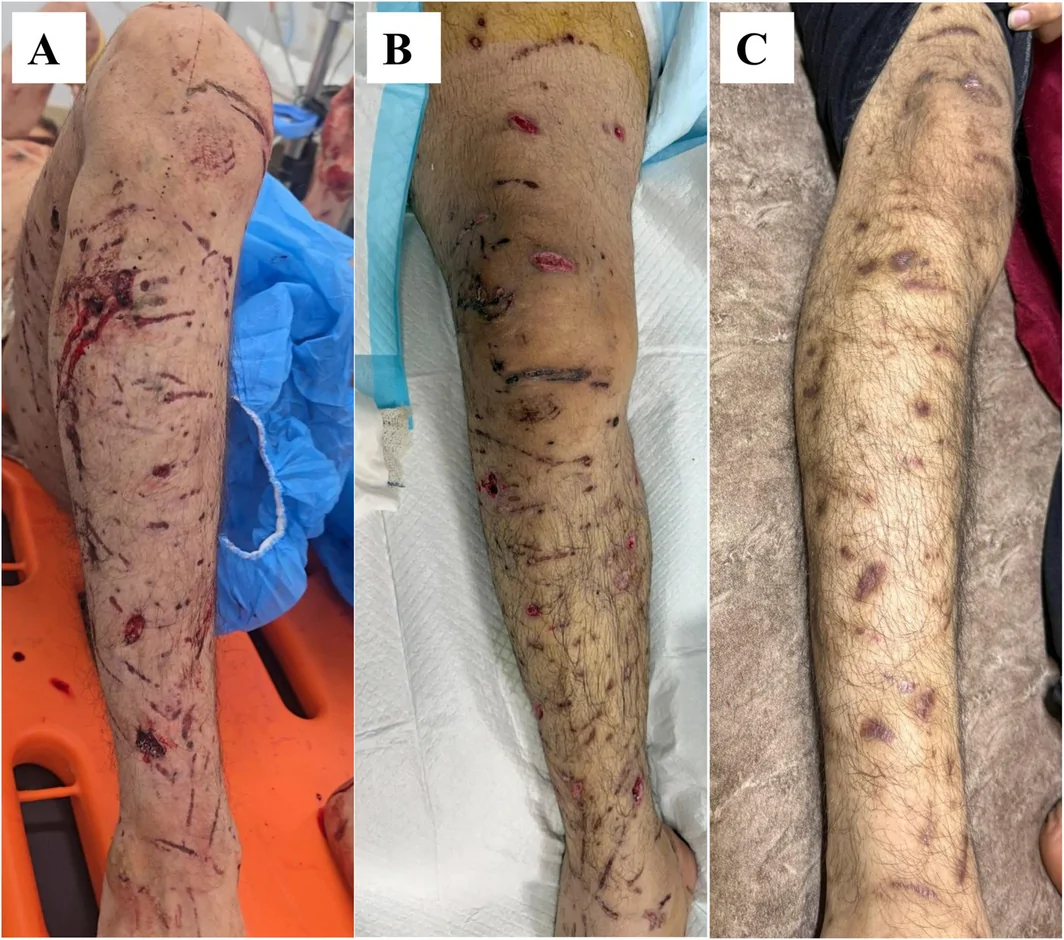
Clinical evolution of lower‐limb dog‐bite wounds. (A) Clinical photograph at presentation showing extensive multifocal lower‐limb dog‐bite wounds, including scattered puncture wounds, linear abrasions/scratch marks, contusions, and irregular lacerations with larger open soft‐tissue wounds. (B) Interval appearance 12 days after presentation, showing multiple healing lower‐limb wounds after irrigation, debridement, and serial wound care, with residual ulcerated areas and granulation tissue among resolving abrasions, puncture wounds, and contusions. (C) Follow‐up photograph 12 weeks after presentation showing marked wound healing, with multiple residual hyperpigmented scars and healed laceration/puncture sites, without visible active ulceration or recurrent soft‐tissue infection.

## 4 Outcome and Follow‐Up

Renal replacement therapy was not required. Throughout the AKI phase, urine output remained adequate and increased to 200–250 mL/h during diuretic therapy, serum potassium remained within the normal range (peak 4.49 mmol/L), and no refractory acidosis or uremic complications developed; therefore, consensus indications for renal replacement therapy were never met [11]. After the initial creatinine peak of 3.48 mg/dL on Day 3, renal function improved before a transient secondary rise began on Day 7 and peaked at 1.94 mg/dL on Day 12, then normalized by Day 14. Vancomycin had already been discontinued within 24 h, and meropenem was replaced by ceftriaxone plus metronidazole on Day 8. Because the secondary rise continued after antimicrobial de‐escalation, coincided with active diuresis and net negative fluid balance, and improved after reduction of diuretic intensity, a predominantly prerenal hemodynamic contribution from over‐diuresis was favored; antibiotic‐associated nephrotoxicity was considered less likely but could not be definitively excluded. The patient gradually improved, with resolution of rhabdomyolysis, coagulopathy, respiratory compromise, and inflammatory abnormalities. Because of profound deconditioning and lower‐limb weakness after the ICU course, he required inpatient rehabilitation before regaining the ability to stand and ambulate. At 12‐week follow‐up, the wounds had healed with residual scars, and he remained well without recurrent renal, hematological, respiratory, or wound‐related complications.

## 5 Discussion

This case demonstrates that extensive dog mauling can produce overlapping traumatic and infectious pathophysiology. The magnitude and early onset of muscle injury supported a major traumatic component, while marked leukocytosis, procalcitonin > 100 ng/mL, and C‐reactive protein 140 mg/L supported a substantial systemic inflammatory response in which bacterial infection could also have contributed. The early posttraumatic inflammatory phase, broad‐spectrum antimicrobial therapy, surgical source control, and transfusion overlapped temporally, making it clinically appropriate to treat the illness as a mixed picture rather than a single dominant mechanism. The AKI was therefore interpreted as multifactorial, with hemorrhagic hypoperfusion, pigment‐mediated injury from rhabdomyolysis, systemic inflammation, and possible infection all as contributing factors.

Despite a peak creatine kinase concentration of 53,960 U/L and creatinine of 3.48 mg/dL, conservative renal management remained appropriate because established indications for renal replacement therapy did not develop. Preserved urine output, a peak potassium of 4.49 mmol/L, and absence of refractory metabolic acidosis or uremic complications supported continued conservative management of AKI. This is consistent with the AAST rhabdomyolysis consensus approach, which emphasizes goal‐directed crystalloid therapy, serial renal and electrolyte monitoring, and renal replacement therapy for conventional clinical indications rather than biochemical severity alone [4, 5, 6]. The later secondary creatinine rise required separate consideration. Vancomycin exposure and concomitant beta‐lactam therapy created a plausible nephrotoxic second‐hit concern, particularly because vancomycin therapeutic drug monitoring was unavailable. However, vancomycin had been stopped within 24 h, meropenem was de‐escalated on Day 8, and the creatinine rise coincided with active negative fluid balance and improved after diuretic intensity was reduced. A hemodynamic prerenal contribution was therefore favored, while drug‐associated nephrotoxicity could not be excluded with certainty.

The case also demonstrates the dynamic nature of trauma/infection‐associated DIC. Despite extreme laboratory derangement, major life‐threatening bleeding was not evident. Transfusion decisions were therefore guided by bleeding status, operative risk, hemodynamics, and serial trends rather than D‐dimer elevation alone. One platelet dose was given when the platelet count reached 18 × 10^9^/L in the setting of extensive traumatic wounds and operative‐site bleeding risk. This illustrates the application of International Society on Thrombosis and Hemostasis guidance, which prioritizes treatment of the underlying cause and reserves platelet support for active bleeding or high‐risk procedures rather than isolated laboratory abnormalities [7, 8].

A major management challenge was the transition from renal‐protective fluid therapy for rhabdomyolysis to de‐resuscitation once fluid overload became clinically evident. On Day 3, pulmonary congestion, pleural effusions, ascites, a noncollapsing inferior vena cava, and dependent oedema developed while renal function remained impaired. Based on serial reassessment using clinical examination, urine output, biochemical trends, chest radiography, and bedside ultrasound, intravenous fluids were reduced on Day 4 and cautious diuresis began, producing a net negative balance of approximately 2.5–3.5 L/day. Respiratory support was weaned as congestion resolved, but the later creatinine rise also illustrates the competing risk of aggressive de‐resuscitation. This balance is consistent with active de‐resuscitation principles emphasizing repeated clinical, biochemical, and ultrasound reassessment rather than a fixed fluid strategy [10].

Published reports of severe systemic complications after dog bites have frequently emphasized infection‐associated sepsis, DIC, AKI, purpura fulminans, amputation, or death [3]. This case adds a complementary teaching point: extensive mauling should trigger simultaneous attention to traumatic muscle injury and infection. Early surveillance for rhabdomyolysis and DIC, prompt wound reassessment and source control, appropriately adjusted antimicrobial therapy, and dynamic fluid stewardship can allow recovery even when severe multiorgan abnormalities coexist and dialysis appears initially conceivable.

## 6 Patient Perspective

The patient recalled the early intensive care period as overwhelming and distressing. He described feeling profoundly weak and unable to move his legs normally, which made even simple tasks such as sitting up or turning in bed impossible without assistance. Standing and walking, he said, were completely out of the question during the first week, and he felt frustrated by his loss of independence. Over time, as his wounds began to heal and his kidney function improved, he started to feel stronger, but he found that regaining the ability to walk was the hardest part of his recovery. With the help of physiotherapy and steady encouragement from his family and the medical team, he gradually progressed from taking a few assisted steps to walking independently. At follow‐up, he was walking without support, had resumed his usual daily activities, and expressed deep gratitude for the care he received.

## Author Contributions

**Rafil A. Bshara:** conceptualization, data curation, investigation, writing – original draft. **Lana Adel Atroshi:** investigation, writing – review and editing. **Zidan Mohammed Abdullah:** conceptualization, writing – review and editing. **Barhast I. Mohamed Salim:** visualization, writing – review and editing. **Gilles Prince:** project administration, writing – review and editing.

## Funding

The authors have nothing to report.

## Ethics Statement

The authors have nothing to report.

## Consent

Written informed consent for publication of this case report, including the clinical photographs, was obtained from the patient's legal guardian in accordance with journal guidelines.

## Conflicts of Interest

The authors declare no conflicts of interest.

## Supporting information


**Supplementary Figure 1** Bedside ultrasound findings during the fluid‐overload phase. (A) Long‐axis hepatic/subxiphoid view demonstrating a dilated inferior vena cava (arrow) with the hepatic vein confluence (asterisk). (B) Right upper quadrant view showing perihepatic free fluid/ascites (arrow). (C) Pelvic view showing free fluid/ascites (arrows) adjacent to the urinary bladder (asterisk). (D) Left lower thoracic view showing a pleural effusion (arrow) above the diaphragm (asterisk). These findings, together with the chest radiograph and physical examination, confirmed multi‐compartment fluid overload and significant third‐spacing during the acute phase of critical illness.

**Supplementary Figure 2** Portable/AP chest radiograph (Day 4) shows increased hazy opacity in the left lower hemithorax with blunting/obscuration of the left costophrenic angle, compatible with a small left pleural effusion with adjacent left basal atelectatic/congestive opacity. No obvious pneumothorax is seen. The right lung is relatively clear. Cardiomediastinal assessment is limited by AP technique and image quality.

## Data Availability

The data that support the findings of this study are available on request from the corresponding author. The data are not publicly available due to privacy or ethical restrictions.
